# Assessing Response Rates and Sleep Disorder Prevalence: Insights from a Propranolol Treatment Study for Infantile Haemangiomas

**DOI:** 10.3390/children11091086

**Published:** 2024-09-04

**Authors:** Francesca Opri, Roberta Opri, Marco Zaffanello, Erika Rigotti

**Affiliations:** 1 Pediatric Clinic, Magalini Hospital, I-37069 Villafranca di Verona, Italy; francesca.opri@aulss9.veneto.it (F.O.); roberta.opri@aulss9.veneto.it (R.O.); 2 Department of Surgical Sciences, Dentistry, Gynecology and Pediatrics, Pediatric Clinic, University of Verona, I-37129 Verona, Italy; 3 Department of Pediatrics, Woman’s & Child’s, University Hospital of Verona, I-37126 Verona, Italy; erika.rigotti@aovr.veneto.it

**Keywords:** children, electronic questionnaire, infantile haemangiomas, infants, propranolol, sleep disturbances

## Abstract

Background: Infantile haemangiomas (IHs) sometimes require treatment with propranolol. Sleep disturbances are the most frequently reported side effects. Monitoring adverse drug events necessitates repeated hospital visits, which can be challenging during a pandemic. Objectives: To explore the effectiveness of a new electronic questionnaire in identifying sleep disturbances related to treatment with propranolol and potential confounding factors. To evaluate the response rate to the questionnaire. To report the proportion of patients on propranolol with sleep disturbances. Methods: In an observational, prospective cohort study, caregivers provided clinical information during ambulatory visits and via an electronic questionnaire after an 8-week treatment course with propranolol and at the time of treatment interruption. Adverse drug reaction reporting forms were assessed for causality. Results: The questionnaire response rate was 91%, and the completion rate was 100%. A total of 59% of patients experienced sleep disturbances during propranolol treatment, which were considered adverse reactions. Sleep disorders were frequent during sleep regression phases and in subjects who fell asleep during physical contact with caregivers or bed-sharing with parents. Conclusion: The application of this questionnaire allows for identifying adverse sleep events associated with propranolol in IHs and potential confounders. Counselling on sleep hygiene is recommended before treatment onset.

## 1. Introduction

### 1.1. Background

Infantile haemangiomas (IHs) represent the most common benign vascular tumour in childhood, with a prevalence of 4–5% [1]. Propranolol, a non-selective beta-blocker, is the first-line treatment for HI [1,2,3]. Although most IHs spontaneously regress, 10–15% of affected patients require systemic treatment with propranolol due to the risk of ulceration or permanent functional or cosmetic damage [2]. Treatment duration typically spans 6 to 12 months, although some cases may require more prolonged therapy [3]. Experience managing hundreds of infants with IHs has shown propranolol to have an excellent safety profile and high tolerability [4,5]. Limited data exist on the utility of β-blockers (atenolol and nadolol) other than propranolol, and on different delivery mechanisms for propranolol [6].

Due to the risk of rare major adverse events, such as hypotension, bradycardia, hypoglycaemia, or bronchospasm, repeated clinical evaluations in a hospital setting are provided [2,7]. Moreover, other widespread minor adverse events, such as sleep disturbances, may increase the need for office visits, placing a considerable managerial burden on the family and potentially exposing children to nosocomial infections [2,7,8]. Furthermore, monitoring patients for any adverse events related to treatment and supporting caregivers was reported as very difficult during the SARS-CoV-2 pandemic [9]. This has created a dramatic impetus towards innovative ways to remotely and effectively monitor patient health status.

### 1.2. Objectives

The study’s primary endpoint was to explore new methods of remote monitoring of the therapy, such as structured questionnaires aimed at the early identification of sleep disturbances as adverse events to treatment and any concomitant factors as potential confounding factors. The study’s secondary endpoint was to report the proportion of treated patients who present with sleep disturbances as drug adverse reactions, i.e., adverse events that can be directly related to drug administration after the causality assessment process conducted by pharmacovigilance experts.

## 2. Materials and Methods

### 2.1. Design and Setting of the Study

This is an observational, uncontrolled, prospective, monocentric, explorative cohort study. The study was conducted in the Paediatric Unit of a tertiary-level hospital (University Hospital of Verona, Veneto Region, Italy) by physicians with expertise in paediatrics and IHs.

### 2.2. Study Population

Participants were enrolled during the first outpatient visit between 1 August 2019 and 1 August 2020. Written informed consent was obtained from the legal representatives of all participants. The local Ethical Committee approved the study (EI-TM Prog. 2792CESC-vers: 2, date of approval 15 July 2020).

All adverse drug reaction (ADR) reporting forms provided by patients on propranolol and their physicians were analysed by experts at the Veneto Region Pharmacovigilance Centre according to Naranjo’s algorithm [10]. The deadline for data analysis was 31 January 2021.

All consecutive patients referred for IHs were considered for recruitment. Inclusion criteria were as follows: age < 2 years; diagnosed with a type of IHs with an indication for systemic treatment with propranolol according to the International Society for the Study of Vascular Anomalies (ISSVA) criteria (i.e., life-threatening IHs, severe functional impairment, ulcerated IHs, permanent aesthetic impairment) [2]. Exclusion criteria were as follows: patient weight < 2000 g at recruitment, heart rate < 80 bpm, non-invasive systemic blood pressure < 50/30 mmHg, atrioventricular block 2–3°, heart failure, phaeochromocytoma, propranolol drug allergy, poor Italian language competence of the patient’s caregivers, lack of electronic instruments such as smartphones or personal computers at the patient’s home.

Since this study was exploratory, the population size was set based on feasibility. Given the patient enrolment capacity for the study, approximately 30 patients were expected to be enrolled over 12 months. Considering a prevalence of sleep disorders of about 30% in the general paediatric population under the age of 24 months [11], and an average of 10% of cases (range 2–20% according to studies) in subjects treated with propranolol for IHs [2], the 95% confidence interval (CI) for the percentage of detectable adverse events, according to Fisher’s exact test probability formula, is 1.6–18.3%.

## 3. Data Collection

### 3.1. Office Visits

Patients’ age, sex, IHs phenotype, sleep habits, and complaints were recorded in medical records at recruitment and each follow-up visit (every 8 weeks and at treatment suspension). Data on personal and family history of diseases and sleep disorders were collected. Children were diagnosed with sleep disturbances if there was a presence, for ≥5 nights per week of prolonged time to fall asleep compared to the norm for that age, and/or ≥3 awakenings per night, and/or prolonged night wakefulness compared to the norm for that age, and/or total duration of sleep (day + night) reduced compared to the norm for that age, and/or excessive daytime sleepiness, and/or increased daytime irritability [12].

### 3.2. Structured Electronic Questionnaire

The electronic interview for the survey is presented as Supplementary Materials. The questionnaire was developed in plain Italian. The first part included anamnestic data, information on pregnancy, the perinatal period, growth, nutrition, and sleep/awake rhythm. Additionally, information about IHs, previous diseases, and family history of significant pathologies was collected. The second part consisted of a previously published sleep quality questionnaire, the Brief Infant Sleep Questionnaire (BISQ), for evaluating infant sleep quality [12].

In order to accurately evaluate any adverse events reported by caregivers, information about potential confounders was also investigated in the third section of the questionnaire. This primarily included non-drug-related factors that might be associated with sleep disorders, such as birth order, the presence of chronic diseases, age during physiological “sleep regression” periods [13], type of feeding, type of care (e.g., exclusive parental care, child care communities, the presence of non-parental caregivers), and sleep hygiene habits (e.g., co-sleeping, bed-sharing, stimuli in the bedroom, methods of falling asleep, interactions with the child during awakenings, and a first-degree family history of sleep disorders).

At recruitment, parents provided their e-mail addresses to the IH-expert physicians involved in the study (F.O., E.R.) to be contacted for the follow-up interview. Additionally, they provided their phone contact details and specified their preferred time intervals for any phone calls (F.O., E.R.). The electronic form of the interview was sent by e-mail, and the caregivers were asked to complete it using a free software application (“Google Forms”, https://docs.google.com/forms/u/0/; accessed date 5 June 2019), which also allowed for automatic data export into an Excel file dataset (Office for Windows, version 365) upon completion of the questionnaire. Within 7 days of sending questionnaires, F.O. and E.R. contacted the parents to identify any significant adverse events or difficulties in completing the interview. Any descriptive notes provided by responders in the electronic interview were used for dataset integration. In discordant opinions between two researchers (F.O., R.O.) regarding the interpretation of notes, a third opinion was sought (E.R.). The questionnaire was administered 8 weeks after treatment and during treatment interruption.

### 3.3. Dataset Management

Clinicians involved in the study (F.O., E.R., R.O.) integrated information from the patient’s medical records with data from the corresponding electronic questionnaires provided by the caregivers. The dataset was then cleaned for duplicates and structural errors. No missing data were found. Finally, the researchers were provided anonymised, person-level data linkage for data analysis.

### 3.4. Statistical Analysis

Chi-square or Fisher’s exact probability tests, Student’s *t*-test, or the Mann–Whitney U test was used as appropriate for categorical variables, quantitative variables with normal distribution, and quantitative variables without normal distribution. The significance threshold was set at *p* < 0.05 for all analyses in the study. Data analysis was performed using SPSS 22.0 version for Windows (SPSS Inc., Chicago, IL, USA).

## 4. Results

**Figure 1:**
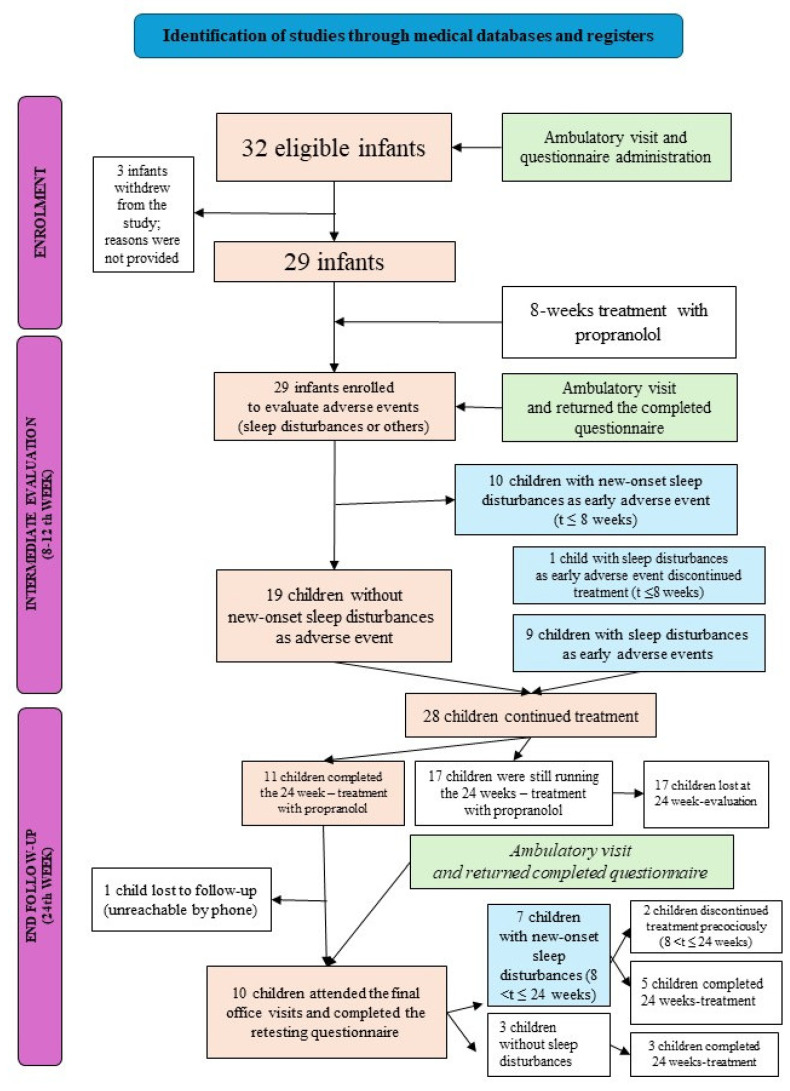
Flow diagram of the study design, patient enrolment, and measures taken. Blue refers to the patients diagnosed with sleep disturbance; pink indicates the children evaluated at each step; green shows the ambulatory visits and questionnaire administration steps.

Thirty-two patients were eligible and accepted enrolment in the study. Subsequently, three patients refused to continue in the study for unknown reasons. The details of the study design, patient enrolment, and measures taken are detailed in Figure 1.

The characteristics of the study population are summarised in Table 1.

The questionnaire was administered to all 32 subjects enrolled in the study (Figure 1). None of the subjects reported sleep disturbances at the enrolment visit. Twenty-two enrolled subjects were native Italian speakers, and ten were non-native Italian speakers. The response rate was 20/22 (91%) among native Italian speakers and 9/10 (90%) among non-native Italian speakers.

Three infants withdrew from the study, and their reasons were not provided.

The education level of the parent completing the questionnaire was represented by a primary school diploma in 2/29 cases (7%), a high school diploma in 12/29 cases (41%), and a degree or postgraduate qualification in 15/29 cases (52%). Among non-native Italian speakers, the education level reported was either a high school diploma (4/9 subjects—44%) or higher (degree or postgraduate qualification in 5/9 cases—56%).

In summary, the response rate obtained from 29 patients was 90.6%. All questionnaires were completed, and all infants started treatment with propranolol.

After 8 weeks of treatment, the 29 enrolled patients (23.6% male; 5.2 ± 4.9 years) were finally seen in the medical office and returned completed questionnaires. Moreover, 19 patients did not experience any sleep-related adverse events. However, one patient withdrew from the study due to a sleep-related adverse event and the subsequent discontinuation of treatment.

At the 24-week follow-up, 10 infants attended the final office visit and completed the questionnaire. Among them, seven children developed new-onset sleep disturbances between 8 weeks and 24 weeks of treatment, and the therapy with propranolol was discontinued prematurely in two of them.

Ten patients returned questionnaires (response rate: 91%), and all completed the interviews (completion rate: 100%). The education level of parents completing the questionnaire was represented by a primary school diploma in 2/10 cases (20%), a high school diploma in 4/10 cases (40%), and a degree or postgraduate qualification in 4/10 cases (40%). Five out of ten subjects (50%) were non-native Italian speakers.

Table 2 summarises the integrated information from office medical records, the electronic interview, and the causality assessment provided for adverse events.

Sleep/awake rhythm disturbances were reported in 10/29 subjects (34%) at the 8–12th-week survey and in 7/10 patients (70%) at the end of follow-up (30 September 2020). According to Naranjo’s algorithm [10], 15 out of 17 children (aged 7.4 ± 6.5 months; 23.5% male) reported sleep disturbances considered potentially related to the treatment, although this was doubtful in 2 very young female patients (Table 2). However, due to the significant negative impact of sleep disturbances on the quality of life for some families, a 2-week trial of drug suspension was required in 2/10 subjects at the 8-week evaluation and in 3/7 patients at subsequent visits. As shown in Table 3, the drug suspension trial was deemed adequate in 1/2 subjects and 2/3 patients, respectively. Among patients who did not undergo a drug suspension trial, one child continued therapy unchanged as the sleep disorder was tolerable after counselling on sleep hygiene and the introduction of melatonin supplementation. Additionally, 11/17 subjects did not require any specific intervention due to the perceived minimal impact of symptoms on their quality of life.

Potential confounders other than propranolol were also evaluated, as shown in Table 4.

First-degree family history of sleep disturbances was considerably more common in subjects with the onset of sleep disorder before 4 months of age (2/3 subjects—67%) compared to those with late-onset (3/14—21%), although this difference was not statistically significant (*p* = 0.110).

## 5. Discussion

The questionnaire used in this study achieved a high response rate (91%), similar among subjects of both Italian and foreign mother tongues, at the initial survey and the remote retesting. This questionnaire’s 100% completion rate indicates that everyone who started it completed it, including retesting. Thus, the questionnaire appeared user-friendly, at least to subjects with a medium level of education, even if they were non-native Italian speakers, and it was not excessively time-consuming for the respondents.

Surveys can be valuable for assessment across various applications and are frequently used in medical education research [14]. According to the literature, the response rate to electronically sent questionnaires is considered the most accurate indicator for assessing the quality of research through surveys and the response rate is considered high when it is above 80% [15].

Regarding sleep disorders, the prevalence detected at outpatient visits was 17%, similar to data reported in the literature (2–20% of cases) [7]. However, following the administration of the computerised anamnestic questionnaire, this prevalence increased to 59%. According to literature data, sleep disturbances are adverse events frequently reported for treatment with propranolol in IHs, and they appear to be even more frequent in this study [16,17]. These data, we argue, are consistent with the possibility of quickly recalling a child’s health information in space and time, which is more comfortable for parents than recalling it in a traditional medical office. It is also interesting to note that the causality assessment for the most sleep-adverse events (15/17) reported by caregivers in this study was “possible” or “probable” based on the Naranjo algorithm. Therefore, the reported “adverse events” should be regarded as “adverse reactions”.

The discontinuation of propranolol due to adverse effects occurred in 13% of the subjects in the present sample, a higher proportion than reported in the literature (2.5% of cases) [17]. This may be consistent, in our opinion, with the strong negative impact of this specific adverse event on the quality of life of all family members.

We found that sleep disorders were significantly more frequent in specific physiological neurodevelopmental stages (“sleep regression” phases) but also in subjects who fell asleep maintaining physical contact with their caregivers, and in those who were bed-sharing with their parents or were actively cradled, breastfed, or moved to their parents’ bed at nocturnal awakenings. Bed-sharing and active parental intervention are well-known risk factors for the development of sleep disorders in children, and several studies have shown that autonomous children falling asleep and falling asleep again in case of awakenings have a longer sleep duration and a lower number of awakenings during the night [18,19,20,21]. Therefore, this study’s data align with literature reports about general risk factors for sleep disorders in the paediatric age. Moreover, some sleep-routine habits may act as facilitating factors for the onset and/or worsening of sleep disturbances during developmental stages (e.g., physiological “sleep regression” phases), independently of any drug therapy. These may be reported by parents as suspected drug adverse events if a new drug was started in the meantime.

However, due to the limited population size of this study and the lack of a formal control group, further investigation is needed to allow for conclusive analyses of any risk factors for the onset of sleep/awake rhythm disturbances as adverse events of treatment with propranolol in IHs.

The clinical relevance of sleep disorders in children and their impact on the quality of life for the whole family unit is widely reported in the literature, noting associations with poor parental physical and mental health, maternal depression, and high levels of family stress [22]. Suppose specific counselling on general sleep habits in children and their expected physiological variations with age was not provided before pharmacological therapy for IHs was introduced. In that case, consideration should be given to the potential onset of sleep/wake disturbances during the treatment course. Parents may interpret these symptoms as ADR. Suppose sleep disturbances are particularly severe, due to high stress levels and the belief in the primary role of the drug in causing these symptoms. In that case, there may be a significant risk of premature treatment discontinuation and poor parental adherence to secondary assessments and specific interventions, such as behavioural therapy or melatonin, as recommended by experts [23].

The administration of an electronic, user-friendly questionnaire before the onset of treatment to identify any risk factors for sleep disturbances and its regular re-administration during the therapy course may support clinicians in reducing sleep disturbances and improving treatment adherence.

Although the sample size is small due to the exploratory nature of the study, we believe that the participants in the study are representative of the target population according to the literature data on patients with IHs, and that all instruments required for data collection after language translation could be highly usable in middle- and high-income countries.

Several studies in the scientific literature have investigated the use of propranolol for the treatment of IHs [2,6,24], mainly investigating the potential side effects of the treatment with propranolol, including sleep disorders such as a decrease in sleep efficiency and an increased number of night-time awakenings compared to the control group at 6 months [25], and irritability with decreased sleep (25.6%) [26]. These studies suggested that sleep disorders are a potential side effect of propranolol treatment for IHs and highlight the importance of counselling on sleep hygiene habits before the start of treatment.

The small number of subjects in the final evaluation might limit the analysis’s power in this exploratory study. Additionally, parents often report sleep disturbances in their otherwise healthy children of the same age, which are frequently classified as behavioural insomnia [27]. This study’s prospective, uncontrolled design enables us to track changes in sleep characteristics in these infants over time.

The strength of our study lies in its ability to offer a thorough and detailed view of the factors affecting infant sleep, using validated tools and contemporary methods for data collection and analysis. This approach could be valuable for future research with different study designs and a wider range of children, potentially providing new insights and recommendations for clinical practice.

Another of the study’s strengths is that it adds to the existing literature using a computerised specific questionnaire to evaluate sleep disorders in infants and toddlers treated with propranolol for IHs. This approach identifies any potential non-drug-related confounding factors for sleep adverse events and helps manage the therapeutic course in these patients.

Additionally, the study highlights the importance of counselling on sleep hygiene habits before beginning treatment to prevent unnecessary interruption of therapy. Overall, it adds to the existing literature by offering new insights and a fresh approach to evaluating the side effects of propranolol treatment for IHs.

## 6. Conclusions

Using this specialised computerised questionnaire, we found a high prevalence of sleep disorders in infants and toddlers treated with propranolol for IHs. Combining the questionnaire results with outpatient evaluations helps identify any non-drug-related factors that might contribute to sleep issues, making it easier to manage propranolol treatment. Additionally, given the significant impact these symptoms can have on families, it is crucial to provide counselling on sleep hygiene before starting treatment This can help prevent unnecessary interruptions in therapy, especially when the treatment has a favourable risk/benefit profile.

## Figures and Tables

**Table 1:** Clinical characteristics of the children (n = 29) enrolled in the study.

Sex (Female/Male)	*n*/*n*	23/6
Age at treatment onset, months	Mean (SD)	5.2 (4.9)
	Median (IQR)	4.2 (1.6–7)
IHs classification, *n*/*n* total (%)	Superficial	14/29 (48)
	Deep	5/29 (17)
	Combined	10/29 (35)
Problems in pregnancy, *n*/*n* total (%)	Twins	3/29 (10.3)
	Preterm birth	7/29 (24)
	Maternal or placental problems	12/29 (41.4)
Indication for propranolol, *n*/*n* total (%)	Functional damage	7/29 (24)
	Ulceration	9/29 (31)
	Risk of cosmetic permanent damage	13/29 (45)

Legend: IHs, infantile haemangiomas; IQR, interquartile range; SD, standard deviation.

**Table 2:** Adverse events were reported during ambulatory visits and through the questionnaire and causality assessment in 17 children with sleep disturbance.

Sex	Age at Treatment Onset (Months)	Ambulatory Visit: Sleep Disturbance	Questionnaire Assessment: Sleep Disturbance	Additional Declarations	Measures Taken	Causality Assessment (Naranjo’s Algorithm)
Female	4.5	Yes	No	≥3 awakenings/night on most nights, average nocturnal sleep onset time ≥20 min.	Definitive discontinuation of pharmacological therapy after 5 months of treatment. The adverse event was unchanged after drug discontinuation.	Possible
Female	24.2	Yes	Yes	≥3 awakenings per night on most nights, average nocturnal sleep onset time ≥20 min.	Definitive discontinuation of pharmacological therapy after 6 months of treatment (IH not yet resolved). The adverse event resolved after drug discontinuation.	Probable
Female	3.5	No	Yes	Mild	None	Probable
Female	11.6	No	Yes	Mild	None	Probable
Female	10.8	No	Yes	≥3 awakenings per night on most nights with an average nocturnal sleep onset time of ≥20 min	None	Probable
Female	3.7	No	Yes	The average nocturnal sleep onset time and the duration of wakefulness during the night are prolonged (30 min and 60 min, respectively), with the presence of sudden awakenings accompanied by crying, daytime irritability, and fatigue.	None	Probable
Female	14.4	Yes	Yes	Daytime irritability	Early and definitive treatment discontinuation (after 1.5 months from initiation), adverse events resolved after drug discontinuations.	Probable
Male	1.8	No	Yes	Daytime irritability	None	Probable
Male	5.8	Yes	Yes	Daytime irritability; sleep disturbance (≥3 awakenings per night, average nocturnal sleep onset time ≥20 min).	None	Probable
Female	2.8	No	Yes	Unconfirmed sleep disturbance and daytime irritability.	None	Doubtful
Female	2.6	No	Yes	Unconfirmed sleep disturbance and daytime irritability.	None	Doubtful
Female	19.9	Yes	Yes	Sleep disturbances with ≥3 awakenings per night.	Melatonin treatment. Continuation of therapy with propranolol at total dose. The adverse event was resolved without drug discontinuation.	Possible
Female	4.9	No	Yes	With hyperhidrosis, the average nocturnal sleep onset time is prolonged to approximately 60 min.	None	Probable
Female	2.1	No	Yes	Excessive daytime sleepiness and irritability.	Temporary discontinuation of treatment. The adverse event was unchanged at drug discontinuation.	Possible
Male	3.6	No	Yes	Excessive daytime sleepiness and irritability.	None	Probable
Female	1.3	No	Yes	Psychomotor agitation during the day, as well as recurrent episodes of bronchospasm; prolonged average nocturnal sleep onset time (approximately 60 min) and ≥3 awakenings per night.	Continuation of therapy at the same dose due to the localisation in the oral cavity with a high risk of complications.	Probable
Male	8.7	Yes	No	Nocturnal sleep disturbance; daytime fatigue and irritability.	Early and definitive discontinuation of therapy after 2.6 months from the start of treatment, with symptoms reported as fully resolved.	Probable

**Table 3:** Sleep disturbances and drug suspension trial effect.

N°	Patient Enrolment		Follow-Up (8 Weeks)		Stop Follow-Up (24 Weeks)	
	O.V.	Q	O.V.	Q	O.V.	Q
Sleep dist. *n*/*n* total (%)	0/29	(-)	3/29 (10%)	10/29 (34%)	3/10 (30%)	7/10 (70%)
Trial effect *n*/*n* total	(-)	(-)	2/10		3/7	
positive			1/10		2/7	
negative			1/10		1/7	
not performed			8/10		4/7	

Legend: (-), not applicable; O.V., office visit; Q, electronic questionnaire; sleep dist., sleep disturbances.

**Table 4:** Potential confounders for sleep disturbances other than propranolol.

Confounders Category	Sleep Disturbances with Possible Confounders	Sleep Disturbances without Possible Confounders	*p*-Value (Chi-Square Test)
	*n*/*n* Total (%)	*n*/*n* Total (%)	
Onset in “sleep regression” phases:	12/17 (71%)	5/12 (42%)	0.110
“4–5 months”	5/17		
“9–11 months”	4/17		
“12–14 months”	2/17		
“24–25 months”	1/17		
Regular time for falling asleep	13/23 (57%)	4/6 (67%)	0.65
To take milk as a preparatory sleep routine	11/22 (50%)	6/7 (86%)	0.09
To fall asleep cradled or in parent’s arms or with physical contact	15/19 (79%)	2/10 (20%)	0.002
To fall asleep with a pacifier	10/15 (67%)	7/14 (50%)	0.36
To fall asleep, take milk	8/15 (53%)	9/14 (64%)	0.54
To fall asleep with electronic objects switched on in the room	4/8 (50%)	13/21 (32%)	0.56
Room-sharing with parents	13/19 (68%)	4/10 (40%)	0.13
Bed-sharing with parents	14/18 (78%)	3/11 (27%)	0.007
Actively cradled or breastfed or moved to parents’ bed at nocturnal awakening	12/16 (75%)	5/13 (38%)	0.046
First-degree family history of sleep disturbances	3/4 (75%)	14/25 (56%)	0.47

## Data Availability

Data are unavailable due to privacy and ethical restrictions.

## Supplementary Materials

The following supporting information can be downloaded at: https://www.mdpi.com/article/10.3390/children11091086/s1, The electronic interview for the survey.

## Abbreviations

ADR	Adverse drug reaction
AE	Adverse event
BISQ	Brief Infant Sleep Questionnaire
IHs	Infantile haemangiomas
IQR	Interquartile range
SD	Standard deviation
