# Supplementary material for: Assessing Response Rates and Sleep Disorder Prevalence: Insights from a Propranolol Treatment Study for Infantile Haemangiomas
Source: Children (Basel). 2024 Sep 4;11(9):1086. doi: 10.3390/children11091086 (PMC11430174; doi:10.3390/children11091086)
Supplement: Supplementary file 1 [file children-11-01086-s001.zip › children-3076230-supplementary.pdf]

Supplementary Materials

Questionnaire in English

We invite you to answer the following questions to allow the project's referring doctors to monitor therapeutic tolerance and any side effects during the pharmacological treatment for infantile haemangiomas. By completing and submitting this questionnaire, you authorise the project's referring doctors to process the minor's personal data, which will be collected and processed in accordance with the current regulations concerning the handling of sensitive data. If you do not wish to provide consent for the collection and use of the requested data, please refrain from submitting the questionnaire.

If desired, you may receive an electronic copy of the completed questionnaire; this copy will be available as soon as the form is submitted.

1. Email address

2. Child's first name. Enter ONLY the first two letters of the name.

3. Child's surname. Enter ONLY the first two letters of the surname.

4. Child's date of birth

5. Child's sex

  - Male

  - Female

6. Who is completing the questionnaire?

  - Mother

  - Father

  - Legal guardian

General Information

7. Were there any problems during the pregnancy? Multiple answers allowed.

  - No. Normal pregnancy.

  - Yes. Threatened miscarriage and/or threatened preterm birth.

  - Yes. Maternal diabetes.

  - Yes. Pregnancy-induced hypertension.

  - Yes. Maternal hypertension.

  - Yes. Placental anomalies.

- Yes. Other problem.

8. Was the child born prematurely (i.e., before 37 weeks of gestation)?

  - Yes
  - No
  - Don’t know

9. Was the child born from a multiple pregnancy?

  - Yes
  - No

10. Does the child have siblings? If yes, please specify the child’s birth order relative to their siblings.

  - The child does NOT have siblings.
  - The child has siblings and is the Firstborn.
  - The child has siblings and is the Second born.
  - The child has siblings and is the Thirdborn.
  - The child has siblings and is the Forthborn.
  - The child has siblings and is the Fifthborn.
  - Other

11. What type of predominant feeding was used?

  - Breastfeeding
  - Bottle-feeding with breast milk
  - Formula feeding

12. If the child was breastfed (either at the breast or from a bottle), at what age was breastfeeding discontinued? Please indicate the age in MONTHS. If the child has always had only formula milk, please leave the answer blank.

13. What was the reason for discontinuing breastfeeding?

  - Insufficient breast milk
  - Poor growth of the child as noted by the attending physician
  - Feeding difficulties (related to the mother or the child)
  - Maternal health reasons
  - Maternal return to work

- Other

14. Has your child already begun weaning?

- Yes
- No

15. At what age did the child begin weaning? Please indicate the age in MONTHS. If the child has not yet begun weaning, please leave the answer blank.

16. If the child has already begun weaning, what weaning method has been used? If the child has not yet begun weaning, please leave the answer blank.

- Baby-led weaning (complementary feeding on demand)
- Traditional weaning

17. Do the parents or siblings of the child suffer from, or have they suffered from, asthma, allergies, or atopic dermatitis?

- Yes
- No
- Don’t know

18. Are the parents and/or other usual caregivers of the child (e.g., grandparents, babysitters) active smokers? Do not consider smoking habits interrupted before the onset of the pregnancy related to the child.

- Yes
- No
- Don’t know

Child's Daily Activities

19. Does the child regularly attend childcare facilities (e.g., Nursery, Playgroup)?

- Yes
- No

20. If the child regularly attends childcare facilities (e.g., Nursery, Playgroup), at what age did they start? Please indicate the age in MONTHS. If the child does not attend childcare facilities, please leave the answer blank.

21. On average, how much time does the child spend in childcare facilities (e.g., Nursery, Playgroup)? Do not include time spent with other caregivers (e.g., grandparents or babysitters) here.

- Up to 6 hours per week
- 7 to 12 hours per week
- 13 to 24 hours per week
- 24 to 36 hours per week
- More than 36 hours per week
- The child does not attend childcare facilities

22. Is the child regularly cared for by other caregivers (e.g., grandparents, babysitters)? Do not consider attendance at Nursery or Playgroup here.

- Yes
- No

23. If the child is regularly cared for by other caregivers (e.g., grandparents, babysitters), at what age did they start? Please indicate the age in MONTHS. If the child is not regularly cared for by caregivers other than the parents, please leave the answer blank.

24. On average, how much time does the child spend with other caregivers (e.g., grandparents or babysitters)? Do not include time spent in childcare facilities (e.g., Nursery, Playgroup) here.

- Up to 6 hours per week
- 7 to 12 hours per week
- 13 to 24 hours per week
- 24 to 36 hours per week
- More than 36 hours per week
- The child is not cared for by other caregivers

Information about the Parents or Legal Guardians

The information requested below is important for understanding how sleep might be influenced by environmental factors. Therefore, it is important that all questions are completed. However, if you do not wish to provide information, you may proceed to the questions in the next section.

25. Mother’s date of birth

26. Mother’s educational qualifications

- School leaving certificate
- High school diploma
- University degree
- Postgraduate qualifications
- Other:

27. Mother’s employment status before the birth of the child.

- Unemployed
- Employed

28. Current employment status of the mother.

- Unemployed
- On maternity leave
- Employed

29. Specify the mother’s current occupation (e.g., "doctor; office worker; teacher…"). If the mother is not currently working, please specify "unemployed" or "on maternity leave" as appropriate.

30. If the mother is currently employed, indicate how long she has been back at work. If the mother is not working, please specify "Currently unemployed" or "Currently on maternity leave."

- Returned to work from the third month after childbirth
- Returned to work from the fourth month after childbirth
- Returned to work from the fifth month after childbirth
- Returned to work from the sixth month after childbirth
- Returned to work from the seventh month after childbirth
- Returned to work from the eighth month after childbirth
- Returned to work from the ninth month after childbirth
- Returned to work from the tenth month after childbirth
- Returned to work from the eleventh month after childbirth
- Returned to work from the twelfth month after childbirth
- Returned to work after the twelfth month after childbirth
- Currently unemployed
- Currently on maternity leave

31. If the mother is currently employed, is her current job the same as her job before childbirth?

- Yes
- No

32. Father's or legal guardian's date of birth

33. Father's or legal guardian's educational qualifications.

- School leaving certificate
- High school diploma
- University degree
- Postgraduate qualifications
- Other:

34. Father's or legal guardian's employment status before the birth of the child.

- Unemployed
- Employed

35. Current employment status of the father or legal guardian.

- Unemployed
- On paternity leave
- Employed

36. Specify the father's or legal guardian's current occupation (e.g., "doctor; office worker; teacher..."). If the father or legal guardian is not currently working, please specify "unemployed" or "on paternity leave" as appropriate.

General Health Status of the Child

37. Does the child currently have, or have they ever had, significant illnesses? Please refer to conditions that have required further investigation, repeated visits, and/or hospital admissions.

- Yes
- No
- I don't know

38. If the child has or has had significant illnesses, please specify which illness. Multiple answers are allowed.

- The child does NOT have any significant illness
- Neurological disorder
- Eye or ear disorder
- Heart and/or circulatory system disorder
- Respiratory system disorder
- Gastrointestinal disorder
- Kidney and/or urinary tract disorder
- Blood disorder
- Skin disorder
- Other

39. If possible, please provide more details about the illness mentioned in the previous answer. Examples: "Tetralogy of Fallot", "bronchopulmonary dysplasia", "epilepsy", "cerebral palsy", "hearing impairment"...

40. Does the child suffer from atopic dermatitis or documented food allergies?

- Yes
- No
- I don't know

General Health Status. Respiratory Disorders

41. Has the child ever had respiratory problems? Consider ONLY symptoms such as persistent cough, bronchospasm, wheezing, accelerated breathing rate compared to usual, difficulty breathing, weak crying, easy fatigue (e.g., difficulty feeding).

- Yes
- No
- I don't know

42. If respiratory problems occurred, did the child also have a cold, fever, or other "flu-like" symptoms at the same time?

- All episodes were WITH accompanying fever and/or flu-like symptoms.
- All episodes were WITHOUT fever or other flu-like symptoms.
- Some episodes were associated with fever or other flu-like symptoms, others were not.
- There were respiratory problems before therapy, but I don’t remember the details.
- There have never been any respiratory problems.

43. If respiratory problems occurred, at what age did they appear? Please express the age in MONTHS. If there were no respiratory problems, please leave the answer blank.

44. If respiratory problems occurred, please indicate HOW MANY episodes of respiratory disturbance (e.g., persistent cough, bronchospasm, wheezing, accelerated breathing rate, difficulty breathing, weak crying, easy fatigue) there were. Report the number of episodes and the considered time frame. Example: "3 episodes in 3 months", "2 episodes in 6 months", "1 episode in 1 year"... If there were no respiratory problems, please leave the answer blank.

45. If present, during which periods of the year did the respiratory symptoms occur? Multiple answers are allowed.

- October to March
- April to June
- July to September
- There were no respiratory problems.

46. If there were respiratory problems, was the child treated with aerosol containing Salbutamol or Ipratropium or puffs?

- Yes
- No
- I don't know
- There have never been any respiratory problems

47. Did the respiratory problems you reported require the child to be admitted to hospital? Consider "admission" as the need to stay in hospital for at least 24 hours.

- There were respiratory problems, but hospital admission was never necessary.
- There were respiratory problems, with ONE hospital admission for this reason.
- There were respiratory problems, with TWO OR MORE hospital admissions for this reason.
- There have never been any respiratory problems.

The Child's Sleep

When answering the following questions, please refer to your child's sleep over the past 2 weeks.

48. How many nights per week are you personally involved in managing your child's evening settling and night-time sleep?

- 0
- 1
- 2
- 3
- 4
- 5
- 6
- 7 nights

49. In PREPARING FOR BEDTIME in the evening, does your child Usually drink milk or breastfeed? Do not consider any breastfeeding that may occur during the subsequent settling phase.

- Yes
- No

50. In which room does your child usually fall asleep in the evening?

- In their own bedroom
- In the parents' bedroom
- In the bedroom of siblings or other relatives
- In another room of the house

51. Where does your child usually fall asleep in the evening?

- In a crib
- In their own bed
- In the parents' bed
- In the parents' room in a separate bed
- In a pram or rocking chair
- In an adult's arms
- Other

52. How does your child usually fall asleep in the evening?

- With the contact of an adult or being rocked
- With an adult in the room, but without contact and without being rocked
- Alone, without an adult in the room

53. Does your child usually drink milk (breast or bottle) WHILE FALLING ASLEEP in the evening? Do not consider any milk intake that occurs as part of the preparation for bedtime.

- Yes
- No

54. Does your child usually fall asleep in the evening with a pacifier?

- Yes
- No

55. When your child falls asleep in the evening, are there usually TVs, tablets, or smartphones on in the same room?

- Yes
- No

56. Who usually puts your child to bed in the evening?

- Always and only the mother
- Usually, the mother
- Both parents, equally
- Always and only the father
- Usually, the father
- Usually, someone else (other than the parents)

57. At what time is your child usually put to bed in the evening? Indicate the time when the light is turned off. Please provide the hour (from 00 to 23) and minutes (from 00 to 59).

- Example: 20:30

58. In a "standard" week, how many times does the child go to bed at the same time? (15-minute tolerance)

- 0
- 1
- 2
- 3
- 4
- 5
- 6
- 7 nights

59. Typically, how difficult is bedtime?

- Very easy
- Quite easy
- Neither easy nor difficult
- Quite difficult
- Very difficult

60. How long does your child usually take to fall asleep? Indicate the time between when the child is put to bed and when they fall asleep. Please always express the time in MINUTES (e.g., 20 minutes; 90 minutes; 150 minutes...).

61. In which room does your child usually sleep for most of the night?

- In their own bedroom
- In the parents' bedroom
- In the bedroom of siblings or other relatives
- In another room of the house

62. Where does your child usually sleep for most of the night?

- In a crib
- In their own bed
- In the parents' bed
- In the parents' room in a separate bed
- In a pram or rocking chair
- In an adult's arms
- Other

63. In which position does your child usually sleep for most of the night?

- On their back
- On their stomach
- On their side

64. How many times does your child wake up during a "typical" night? Please indicate the TOTAL NUMBER of awakenings per night.

65. When your child wakes up during the night, what is usually your reaction? Multiple answers allowed.

- I let the child cry and fall back asleep on their own
- I wait a few minutes without intervening to see if the child falls back asleep
- I comfort the child by talking to them, but I do not pick them up
- I sing to the child
- I offer a pacifier
- I offer a bottle
- I maintain physical contact with the child but do not pick them up
- I pick the child up but put them back in bed while still awake
- I pick the child up and rock them until they fall back asleep
- I lie down in bed with the child
- I breastfeed the child until they fall back asleep
- I change the nappy
- I play with the child, watch TV with them, or use a tablet or smartphone together
- I bring the child into my bed
- None of these options

66. Who usually intervenes when the child wakes up at night?

- Always and only the mother
- Usually the mother
- Both parents, equally
- Always and only the father
- Usually the father
- Usually someone else (other than the parents)

67. How long, IN TOTAL, does your child stay awake during the night? Please always express the time in MINUTES (e.g., 20 minutes; 90 minutes; 150 minutes...). Example: If the child wakes up 2 times for 15 minutes each time, indicate the total time as "30 minutes".

68. For how long can your child sleep without waking up during the night? Indicate the MAXIMUM duration of sleep, expressed in MINUTES (e.g., 20 minutes; 90 minutes; 150 minutes...). Example: If the child wakes up after a maximum of 30 minutes of sleep, indicate the total time as "30 minutes".

69. Does your child snore during sleep?

- Never (only if they are ill or have a cold)

- Only occasionally
- Less than 3 times a week
- 3 times a week or more

70. What time does your child wake up in the morning? Please provide the hour (from 00 to 23) and minutes (from 00 to 59).

- Example: 08:30

71. How long, IN TOTAL, does your child sleep during a "typical" night? Add up the duration of all sleep periods from falling asleep to morning wake-up and provide the time in HOURS. Example: Indicate the total time as "6 hours".

72. Where does your child usually wake up in the morning?

- In a crib
- In their own bed
- In the parents' bed
- In the parents' room in a separate bed
- In a pram or rocking chair
- In an adult's arms
- Other

73. Does your child sleep well during the night?

- Yes. They sleep very well.
- Yes. They sleep well.
- Yes. They sleep well.
- No. They sleep poorly.
- No. They sleep very poorly.

74. How would you describe your child's mood when they wake up in the morning?

- In excellent spirits
- In good spirits
- Normal
- Rather bad-tempered
- In a very bad mood

75. On a "standard" day, how many naps does your child Usually have during the DAY? Indicate the TOTAL NUMBER of naps observed between morning wake-up and bedtime.

76. How long, IN TOTAL, does your child nap during the DAY on a "standard" day? Add up the duration of all daytime naps. Please express the total time in MINUTES (e.g., 20 minutes; 90 minutes; 150 minutes...).

77. Do you consider your child's sleep to be a problem?

- No, it is not a problem at all.
- Yes, but it is a minor problem.
- Yes, it is a small problem.
- Yes, it is a moderate problem.
- Yes, it is a serious problem.

78. How confident do you feel in managing your child's sleep?

- Very confident
- Quite confident
- Average
- Quite uncertain
- Very uncertain

Family Sleep Patterns

79. Do the parents or siblings of the child have sleep disorders?

- Yes
- No
- Don't know

80. If you answered "Yes" to the previous question, please specify the type of sleep disorder being reported. Multiple answers allowed.

- Difficulty falling asleep
- Restless Legs Syndrome
- Frequent awakenings during the night
- Nightmares or vivid dreams
- Sleepwalking
- Sleep talking

- Teeth grinding (bruxism)
- Other:

81. What time do you usually go to bed in the evening? Please provide the hour (from 00 to 23) and minutes (from 00 to 59).

82. What time do you usually wake up in the morning? Please provide the hour (from 00 to 23) and minutes (from 00 to 59).

83. Upon waking in the morning, do you usually feel sufficiently rested?

- Yes
- No

Ongoing Medication

84. Does the child take medication as a chronic therapy? Please do not consider vitamin supplements (e.g., vitamin D, vitamin K).

- Yes
- No

85. If the child is on chronic medication, has this therapy been ongoing for more than 10 days?

- Yes, the child has been on medication for more than 10 days
- Yes, the child is on medication, but the therapy has been ongoing for less than 10 days
- The child is NOT on any chronic therapy

86. If the child is on chronic medication, does the therapy include propranolol?

- Yes
- No

Propranolol Therapy

87. At what time does your child usually take the first daily dose of the medication? Please provide the hour (from 00 to 23) and minutes (from 00 to 59).

88. At what time does your child usually take the second daily dose of the medication? Please provide the hour (from 00 to 23) and minutes (from 00 to 59).

89. Regardless of the condition for which the child is taking the medication, do you believe that anything has changed after starting propranolol therapy? Do not consider any changes related to the underlying condition.

- No, I have not noticed any changes.
- Yes, there have been minor changes.
- Yes, there have been significant changes.
- Don't know

After Therapy... Has Anything Changed?

90. If you have noticed changes with the therapy, and regardless of any modifications to the underlying condition, what do you believe has changed in your child after starting propranolol therapy? Multiple answers allowed.

- No changes of any kind have occurred.
- Episodes of bronchospasm and/or other respiratory disorders have occurred.
- Episodes of weakness associated with excessive sweating and/or reduced responsiveness have occurred.
- Episodes of diarrhoea without vomiting and/or fever have occurred.
- Changes in sleep (night-time and/or daytime) have occurred.
- Irritability and/or agitation during the day have occurred.
- Excessive daytime sleepiness has occurred.
- Other type of change

91. If you selected "Other type of change" in the previous question, please specify what changes you have observed.

92. If you have observed changes after starting propranolol therapy, please indicate the age of the child in MONTHS when these changes occurred. If no changes have been observed, please leave this blank.

93. In relation to any changes you have reported, has the therapy been adjusted?

- No, the therapy has not been adjusted as I have not observed any issues.
- No, the therapy has not been adjusted as I did not think it necessary to report the changes to the doctor.
- No, the therapy has not been adjusted despite reporting the changes.
- Yes, the therapy has been permanently discontinued.
- Yes, the therapy has been temporarily discontinued and then resumed.

After Therapy... Respiratory Disorders

94. Following the introduction of propranolol therapy, have you noticed any changes regarding respiratory disorders? Consider ONLY symptoms such as persistent cough, bronchospasm, wheezing, accelerated breathing rate compared to usual, difficulty breathing, weak crying, easy fatigue (e.g., difficulty feeding).

- There have never been any respiratory disorders (neither in the past nor after therapy). → Proceed to question 103.

- New respiratory disorders have appeared that were not present before.

- There were already respiratory disorders, and these have remained unchanged after therapy.

- There were already respiratory disorders, and these have worsened after therapy.

- There were already respiratory disorders, and these have improved after therapy.

95. If respiratory problems occurred after propranolol therapy, was the child simultaneously experiencing a cold, fever, or other flu-like symptoms?

- Respiratory problems occurred after therapy, but I cannot recall their characteristics.

- Some episodes were associated with fever or other flu-like symptoms, while others were not.

- All episodes were WITHOUT fever or other flu-like symptoms.

- All episodes were WITH concurrent fever and/or flu-like symptoms.

96. If respiratory problems occurred, please indicate HOW MANY episodes of respiratory disorder (e.g., persistent cough, bronchospasm, wheezing, accelerated breathing rate, difficulty breathing, weak crying, easy fatigue) occurred. Provide the number of episodes and the time period considered. Example: "3 episodes in 3 months", "2 episodes in 6 months", "1 episode in 1 year"...

97. If the reported respiratory disorders appeared de novo or worsened after starting therapy, how long after the introduction of therapy do you believe these changes occurred? Please specify the time in WEEKS.

98. Do you believe that your child's respiratory disorder represents a problem?

- My child has some respiratory disorders, but it is not a problem at all.

- My child has some respiratory disorders, but it is of little concern.

- My child has some respiratory disorders, but it is a minor problem.

- My child has some respiratory disorders, and it is a moderate problem.

- My child has some respiratory disorders, and it is a serious problem.

99. If respiratory disorders were reported after the introduction of therapy, was there a need for more frequent use of inhaled medications containing Salbutamol) or Ipratropium or puffs COMPARED TO THE PERIOD BEFORE PROPRANOLOL THERAPY?

- Yes, there are respiratory disorders, but the use of these medications has remained unchanged compared to the past.

- Yes, there are respiratory disorders, and these medications have been used more frequently compared to the past.
- Yes, there are respiratory disorders, and these medications have been used less frequently compared to the past.

100. Have the respiratory problems you reported after propranolol therapy required hospitalisation of the child? Consider "hospitalisation" as the need to stay in hospital for at least 24 hours.

- There were respiratory problems after the introduction of therapy, but NO hospitalisation for this reason.
- There were respiratory problems after the introduction of therapy, with ONE hospitalisation for this reason.
- TWO OR MORE hospitalisations were required due to respiratory disorders that occurred after propranolol therapy.

101. In relation to any respiratory disorders reported, has the propranolol therapy been adjusted?

- Yes, the therapy has been permanently discontinued.
- Yes, the therapy was temporarily discontinued and then resumed.
- No, the therapy has not been adjusted.

102. If the therapy was modified due to interference with the child's respiratory disorders, do you believe that the therapeutic modification had a noticeable effect on these disorders in the subsequent period? If no modifications were made to the therapy, please leave this blank.

- The respiratory disorder did not change after the therapy modification.
- The respiratory disorder improved after the therapy modification.
- The respiratory disorder worsened after the therapy modification.
- Don't know
- No therapy modifications were made

After Therapy... Sleep

103. Following the introduction of propranolol therapy, have you noticed any specific changes regarding your child's sleep?

- Yes
- No
- Don't know

104. If you have observed changes in your child's sleep following continuous propranolol therapy, please indicate which disturbances you have noticed. Multiple responses are allowed.

- I have not noticed any changes.
- There has been irritability and/or agitation during the daytime.
- There has been irritability and/or agitation during the night-time.
- There have been episodes of inconsolable crying during the night-time.
- There have been nightmares and/or sudden awakenings with crying.
- There has been excessive daytime sleepiness and/or fatigue.
- There has been difficulty falling asleep during the daytime.
- There has been difficulty falling asleep during the night-time.

105. If you have observed changes in your child's sleep following propranolol therapy, please indicate if any modifications were made to the therapy.

- No, the therapy was not modified because I did not notice any changes.
- No, the therapy was not modified because I did not consider it necessary to report this change to the doctor.
- No, the therapy was not modified despite reporting this change to the doctor.
- Yes, the therapy was temporarily discontinued and then resumed.
- Yes, the therapy was permanently discontinued.

106. If the therapy was modified due to interference with your child's sleep, do you believe that the therapeutic modification had a noticeable effect on sleep in the subsequent period?

- Yes. Sleep improved after the modification of therapy.
- No. Sleep did not change after the modification of therapy.
- Don't know
- The therapy was not modified.

Questionnaire in Italian (original)

La invitiamo a rispondere alle seguenti domande per consentire ai Medici referenti del progetto un monitoraggio della tolleranza terapeutica e di eventuali effetti collaterali in corso di trattamento farmacologico per emangiomi infantili.

Completando ed inviando il questionario, Lei autorizza i Medici referenti del progetto al trattamento dei dati personali del minore, che verranno raccolti ed elaborati nel rispetto delle vigenti norme relative al trattamento dei dati sensibili. Qualora non desiderasse fornire il consenso alla raccolta ed all'utilizzo dei dati richiesti, preghiamo di non procedere con l'invio del questionario.

Se lo desidera potrà ricevere copia in formato elettronico del questionario da Lei completato; tale copia sarà disponibile non appena effettuato l'invio del form.

1. Indirizzo email.

2. Nome del bambino. Indicare SOLO le prime due lettere del nome.

3. Cognome del bambino. Indicare SOLO le prime due lettere del cognome.

4. Data di nascita del bambino

5. Sesso del bambino

- Maschio
- Femmina

6. Chi compila il questionario?

- Madre
- Padre
- Tutore legale

Informazioni generali

7. Vi sono stati problemi nel corso della gravidanza? Sono ammesse più risposte.

- No. Gravidanza regolare.
- Sì. Minacce d'aborto e/o minacce di parto prematuro.
- Sì. Diabete materno.
- Sì. Gestosi gravidica.
- Sì. Ipertensione arteriosa materna.
- Sì. Anomalie della placenta.
- Sì. Altro problema.

8. Il bambino è nato prematuro (ovvero prima delle 37 settimane di gestazione)?

- Sì
- No
- Non so

9. Il bambino è nato da gravidanza gemellare?

- Sì
- No

10. Il bambino ha fratelli? Se sì, indichi l'ordine di nascita del bambino rispetto ai fratelli.

- ☐ Il bambino NON ha fratelli.
- ☐ Il bambino ha fratelli ed è Primogenito.
- ☐ Il bambino ha fratelli ed è Secondogenito.
- ☐ Il bambino ha fratelli ed è Terzogenito.
- ☐ Il bambino ha fratelli ed è Quartogenito.
- ☐ Il bambino ha fratelli ed è Quintogenito.
- ☐ altro

11. Quale tipo di allattamento prevalente è stato effettuato?

- ☐ con latte materno al seno
- ☐ con latte materno al biberon
- ☐ con latte artificiale

12. Se il bambino è stato allattato con latte materno (al seno o al biberon), a che età è stato sospeso l'allattamento materno prevalente? Indicare l'età in MESI. Se il bambino ha sempre assunto solo latte artificiale, si prega di lasciare la risposta in bianco.

13. Per quale motivo è stato sospeso l'allattamento materno?'

- ☐ latte materno insufficiente
- ☐ scarsa crescita del bambino rilevata dal Medico curante
- ☐ difficoltà all'allattamento (per motivi correlati alla madre o al bambino)
- ☐ motivazione di salute della madre
- ☐ rientro della madre al lavoro
- ☐ altro

14. Il Suo bambino ha già intrapreso lo svezzamento?

- ☐ Sì
- ☐ No

15. A che età il bambino ha intrapreso lo svezzamento? Indicare l'età in MESI. Se il bambino non ha ancora intrapreso lo svezzamento, si prega di lasciare la risposta in bianco.

16. Se il bambino ha già intrapreso lo svezzamento, quale modalità di svezzamento è stata adottata? Se il bambino non ha ancora intrapreso lo svezzamento, si prega di lasciare la risposta in bianco.

- ☐ autosvezzamento (alimentazione complementare a richiesta)
- ☐ svezzamento tradizionale

17. I genitori o i fratelli del bambino soffrono, o hanno sofferto in passato, di asma, allergie o dermatite atopica?

- ☐ Sì
- ☐ No

- Non so

18. I genitori e/o gli altri care-givers abituali del bambino (nonni, baby-sitter...) sono fumatori attivi? Non considerare qui l'eventuale abitudine al fumo interrotta prima dell'insorgere della gravidanza relativa al bambino.

- Sì
- No
- Non so

Attività quotidiane del bambino

19. Il bambino frequenta regolarmente comunità infantili (Asilo Nido, Baby-parking)?

- Sì
- No

20. Se il bambino frequenta abitualmente comunità infantili (Asilo Nido, Baby-parking), a che età ha iniziato tale frequenza? Indicare l'età in MESI. Qualora il bambino non fosse inserito in comunità infantili, si prega di lasciare la risposta in bianco.

21. Indicare quanto tempo, in media, il bambino trascorre in comunità infantili (Asilo Nido, Baby-parking). Non conteggiare qui l'eventuale tempo trascorso dal bambino con altri care-givers (nonni o baby-sitter).

- Fino a 6 ore a settimana
- Da 7 a 12 ore a settimana
- Da 13 a 24 ore a settimana
- Da 24 a 36 ore a settimana
- Più di 36 ore a settimana
- Il bambino non è inserito in comunità infantili

22. Il bambino è abitualmente affidato ad altri caregivers (nonni, baby-sitter)? Non considerare qui la frequenza in Asilo Nido o Baby-parking.

- Sì
- No

23. Se il bambino è affidato abitualmente ad altri care-givers (nonni, baby-sitter...), a che età ha iniziato tale frequenza? Indicare l'età in MESI. Qualora il bambino non fosse accudito regolarmente da care-givers diversi dai genitori, si prega di lasciare la risposta in bianco.

24. Indicare quanto tempo, in media, il bambino trascorre con altri care-givers (nonni o baby-sitter). Non conteggiare qui l'eventuale tempo trascorso dal bambino in comunità infantili (Asilo Nido, Baby-parking).

- FIno a 6 ore a settimana
- Da 7 a 12 ore a settimana
- Da 13 a 24 ore a settimana

- Da 24 a 36 ore a settimana
- Più di 36 ore a settimana
- Il bambino non è affidato ad altri care-givers

Dati relativi ai genitori o tutori legali.

Le informazioni di seguito richieste sono importanti per comprendere il modo in cui il sonno possa essere influenzato da situazioni ambientali. È pertanto importante che tutti i quesiti siano completati. Tuttavia, qualora non desiderasse fornire informazioni in merito, potrà procedere con i quesiti riportati nella sezione successiva.

25. Data di nascita della madre

26. Titolo di studio della madre

- Diploma di scuola dell'obbligo
- Diploma di scuola superiore
- Laurea
- Titolo di studio post-laurea
- Altro:

27. Posizione lavorativa della madre prima della nascita del bambino.

- Priva di occupazione
- Occupata lavorativamente

28. Attuale posizione lavorativa della madre.

- priva di occupazione
- in congedo per maternità
- occupata lavorativamente

29. Specificare l'attuale occupazione lavorativa della madre (es. "medico; impiegata; insegnante..."). Se la madre non è attiva lavorativamente, specificare per favore "priva di occupazione" o "in congedo per maternità", come opportuno.

30. Se la madre risulta attualmente occupata, indicare da quanto tempo ha ripreso l'attività lavorativa. Se la madre non lavora, indicare "Attualmente priva di occupazione" o "Attualmente in congedo per maternità".

- Lavoro dal terzo mese dopo il parto
- Lavoro dal quarto mese dopo il parto
- Lavoro dal quinto mese dopo il parto
- Lavoro dal sesto mese dopo il parto
- Lavoro dal settimo mese dopo il parto
- Lavoro dall'ottavo mese dopo il parto
- Lavoro dal nono mese dopo il parto
- Lavoro dal decimo mese dopo il parto

- Lavoro dall'undicesimo mese dopo il parto
- Lavoro dal dodicesimo mese dopo il parto
- Lavoro. Ho ripreso dopo il dodicesimo mese dopo il parto
- Attualmente priva di occupazione
- Attualmente in congedo per maternità

31. Se la madre risulta attualmente occupata, la tipologia dell'attività lavorativa in corso è la medesima di quella antecedente il parto?

- Sì
- No

32. Data di nascita del padre o tutore legale

33. Titolo di studio del padre o tutore legale.

- Diploma di scuola dell'obbligo
- Diploma di scuola superiore
- Laurea
- Titolo di studio post-laurea
- Altro:

34. Posizione lavorativa del padre o tutore legale prima della nascita del bambino.

- Privo di occupazione
- Occupato lavorativamente

35. Attuale posizione lavorativa del padre o tutore legale.

- privo di occupazione
- in congedo per paternità
- occupato lavorativamente

36. Specificare l'attuale occupazione lavorativa del padre o tutore legale (es. "medico; impiegata; insegnante..."). Se il padre o tutore legale non è attivo lavorativamente, specificare per favore "privo di occupazione" o "in congedo per paternità", come opportuno.

Stato di salute generale del bambino

37. Il bambino ha attualmente, o ha avuto in passato, malattie importanti? Si riferisca qui a condizioni che abbiano richiesto indagini di approfondimento, visite ripetute e/o ricoveri ospedalieri.

- Sì
- No
- Non so

38. Se il bambino ha o ha avuto malattie importanti, indichi per favore di quale malattia si tratti. Sono ammesse più risposte.

- ☐ Il bambino NON ha alcuna malattia importante
- ☐ Malattia del sistema nervoso
- ☐ Malattia della vista o dell'udito
- ☐ Malattia del cuore e/o dell'apparato circolatorio
- ☐ Malattia dell'apparato respiratorio
- ☐ Malattia dell'apparato gastroenterico
- ☐ Malattia dei reni e/o delle vie urinarie
- ☐ Malattia del sangue
- ☐ Malattia della pelle
- ☐ altro

39. Specifichi più in dettaglio, se Le è possibile, il tipo di malattia cui si riferisce nella risposta precedente. Esempi: "Tetralogia di Fallot", "broncodisplasia del prematuro", "epilessia", "paralisi cerebrale", "sordità"...

40. Il bambino soffre di dermatite atopica o allergie alimentari documentate?

- ☐ Sì
- ☐ No
- ☐ Non so

Stato di salute generale. Disturbi respiratori

41. Il bambino ha mai avuto problemi respiratori? Considerare SOLO sintomi come tosse persistente, broncospasmo, respiro sibilante, ritmo respiratorio accelerato rispetto all'abituale, difficoltà respiratoria, pianto debole, facile affaticamento (ad esempio, con difficoltà ad alimentarsi).

- ☐ Sì
- ☐ No
- ☐ Non so

42. Se si sono verificati i problemi respiratori sopracitati, il bambino presentava contemporaneamente raffreddore, febbre o altri sintomi "simil-influenzali"?

- ☐ Tutti gli episodi erano CON contestuale febbre e/o sintomi simil-influenzali.
- ☐ Tutti gli episodi erano SENZA febbre o altri sintomi simil-inflenzali.
- ☐ Alcune volte gli episodi erano associati a febbre o altri sintomi simil-influenzali, altri no.
- ☐ Ci sono stati problemi respiratori prima della terapia, ma non ne ricordo le caratteristiche.
- ☐ Non ci sono mai stati problemi respiratori.

43. Se si sono verificati problemi respiratori, a che età del bambino si sono presentati?' Esprimere l'età in MESI. Se non si sono rilevati problemi respiratori, si prega di lasciare la risposta in bianco.

44. Se si sono verificati problemi respiratori, indichi per favore QUANTI episodi di disturbo respiratorio (es. tosse persistente, broncospasmo, respiro sibilante, ritmo respiratorio accelerato, difficoltà respiratoria, pianto debole, facile affaticamento) si sono verificati. RIportare il numero di episodi e l'intervallo temporale considerato. Esempio: "3 episodi in 3 mesi", "2 episodi in 6 mesi", "1 episodio in 1 anno"... Se non si sono rilevati problemi respiratori, si prega di lasciare la risposta in bianco.

45. Se presenti, in quali periodi dell'anno si verificavano i sintomi respiratori? Sono ammesse più risposte.

- Da ottobre a marzo
- Da aprile a giugno
- Da luglio a settembre
- Non ci sono stati problemi respiratori.

46. Se ci sono stati problemi respiratori, il Medico ha trattato questi disturbi con aerosol contenente Salbutamolo o Ipratroprio oppure puff?

- Sì
- No
- Non so
- Non ci sono mai stati problemi respiratori

47. I problemi respiratori che Lei ha segnalato hanno richiesto ricovero del bambino in ospedale? Considerare come "ricovero" la necessità di permanere in ospedale per almeno 24 ore.

- Ci sono stati disturbi respiratori ma NON sono mai stati necessari ricoveri per questo motivo.
- Ci sono stati disturbi respiratori, con UN SOLO ricovero per questo motivo.
- Ci sono stati disturbi respiratori, con DUE O PIU' ricoveri per questo motivo
- Non ci sono mai stati problemi respiratori.

Il sonno del bambino

Nel rispondere alle domande di seguito riportate, La preghiamo di fare riferimento al sonno del Suo bambino nelle ultime 2 settimane.

48. Quanti notti a settimana Lei è coinvolto in prima persona nella gestione dell'addormentamento serale e del sonno notturno del bambino?

0 1 2 3 4 5 6 7 notti

49. Nel PREPARARSI PER ANDARE A DORMIRE la sera, il Suo bambino generalmente beve latte o viene allattato al seno? Non considerare in questa risposta l'eventuale allattamento che viene proposto nella successiva fase di addormentamento.

- Sì
- No

50. In quale stanza si addormenta abitualmente il Suo bambino la sera?

- nella propria stanza da letto
- nella stanza da letto dei genitori
- nella stanza da letto di fratelli o di altri parenti
- in un'altra stanza della casa

51. Dove si addormenta abitualmente il Suo bambino la sera?

- nella culla
- nel proprio letto
- nel letto dei genitori
- nella stanza dei genitori in un letto separato
- nel passeggino o nella seggiolina a dondolo
- in braccio ad un adulto
- altro

52. In che modo si addormenta abitualmente il Suo bambino la sera?

- col contatto di un adulto o cullato
- con un adulto nella stanza, ma senza contatto di un adulto e senza essere cullato
- da solo, senza un adulto nella stanza

53. Il Suo bambino abitualmente beve latte (al seno o al biberon) MENTRE SI ADDORMENTA la sera? Non considerare in questa risposta l'eventuale allattamento proposto in una fase precedente (es. come preparativo per andare a dormire).

- Sì
- No

54. Il Suo bambino si addormenta abitualmente la sera con il ciuccio?

- Sì
- No

55. Quando il Suo bambino si addormenta la sera, sono abitualmente accesi nella stessa stanza TV, tablet o smartphone?

- Sì
- No

56. Chi mette abitualmente a letto il Suo bambino la sera?

- Sempre e solo la madre
- Generalmente la madre
- Entrambi i genitori, in pari misura
- Sempre e solo il padre
- Generalmente il padre
- Generalmente qualcun altro (diverso dai genitori)

57. A che ora viene messo abitualmente a letto il Suo bambino la sera? Indicare l'ora in cui si spegne la luce. Indicare ora (da 00 a 23) e minuti (da 00 a 59).

Esempio: 08:30

58. In una settimana "standard", quante volte il bambino viene coricato alla stessa ora? (tolleranza di 15 minuti in più o meno)

0 1 2 3 4 5 6 7 notti

59. Abitualmente, quanto è difficile il momento di andare a letto?

- Molto facile
- Abbastanza facile
- Nè facile nè difficile
- Abbastanza difficile
- Molto difficile

60. Quanto tempo impiega abitualmente il Suo bambino per addormentarsi? Indicare il tempo trascorso tra il momento in cui il bambino viene messo a letto e quello in cui il bambino si addormenta. Si prega di esprimere SEMPRE il tempo in MINUTI (es. 20 minuti; 90 minuti; 150 minuti...).

61. In quale stanza dorme abitualmente il Suo bambino per la maggior parte della notte?

- nella propria stanza da letto
- nella stanza da letto dei genitori
- nella stanza da letto di fratelli odi altri parenti
- in un'altra stanza della casa

62. Dove dorme abitualmente il Suo bambino per la maggior parte della notte?

- nella culla
- nel proprio letto
- nel letto dei genitori
- nella stanza dei genitori in un letto separato
- nel passeggino o nella seggiolina a dondolo
- in braccio ad un adulto
- altro

63. In quale posizione dorme abitualmente il Suo bambino per la maggior parte della notte?

- supino (a pancia in su)
- prono (a pancia in giù)
- sul fianco

64. Quante volte si risveglia il Suo bambino nel corso di una notte "tipica"? Indichi il NUMERO totale di risvegli per singola notte.

65. Quando il Suo bambino si risveglia nel corso della notte, qual è normalmente la Sua reazione? Sono ammesse più risposte.

- Lascio che il bambino pianga e che si riaddormenti da solo
- Attendo alcuni minuti senza intervenire, per vedere se il bambino si riaddormenta
- Conforto il bambino parlandogli, ma non lo prendo in braccio
- Canto al bambino
- Offro l ciuccio
- Offro il biberon
- Mantengo un contatto fisico col bambino ma non lo prendo in braccio
- Prendo il bambino in braccio ma lo rimetto nel letto quando è ancora sveglio
- Prendo il bambino in braccio e lo cullo fino a quando si riaddormenta.
- Mi distendo nel letto assieme al bambino
- Allatto il bambino fino al riaddormentamento
- Cambio il pannolino
- Gioco col bambino, guardo la TV con lui o usiamo insieme il tablet o lo smartphone
- Porto il bambino nel mio letto
- Nessuna di queste opzioni

66. Chi interviene abitualmente quando il bambino si risveglia la notte?

- Sempre e solo la madre
- Generalmente la madre
- Entrambi i genitori, in pari misura
- Sempre e solo il padre
- Generalmente il padre
- Generalmente qualcun altro (diverso dai genitori)

67. Quanto tempo, IN TOTALE, il Suo bambino rimane sveglio durante la notte? Si prega di esprimere SEMPRE il tempo in MINUTI (es. 20 minuti; 90 minuti; 150 minuti...). Es. Se il bambino si risveglia 2 volte per un tempo di 15 minuti in ciascuna occasione, indicare come tempo totale "30 minuti".

68. Per quanto tempo il Suo bambino può dormire senza svegliarsi nel corso della notte? Indichi la durata MASSIMA del sonno, espressa in MINUTI (es. 20 minuti; 90 minuti; 150 minuti...). Es. Se il bambino si risveglia dopo massimo 30 minuti di sonno, indicare come tempo totale "30 minuti".

- 69. Il Suo bambino russa durante il sonno?
- Mai (solo se è malato o raffreddato)
- Solo occasionalmente
- Meno di 3 volte a settimana
- 3 volte a settimana o più

70. A che ora si sveglia il Suo bambino il mattino? Indicare ora (da 00 a 23) e minuti (da 00 a 59).

Esempio: 08:30

71. Quanto tempo, COMPLESSIVAMENTE, il Suo bambino dorme durante una notte "tipica"? Sommare la durata di tutti i periodi di sonno tra l'addormentamento ed il risveglio del mattino ed indicare il tempo espresso in ORE. Es. Indicare come tempo totale "6 ore".

72. Dove si risveglia abitualmente il Suo bambino il mattino?

- nella culla
- nel proprio letto
- nel letto dei genitori
- nella stanza dei genitori in un letto separato
- nel passeggino o nella seggiolina a dondolo
- in braccio ad un adulto
- altro

73. Il bambino dorme bene durante la notte?

- Sì. Dorme molto bene.
- Sì. Dorme bene.
- Sì. Dorme abbastanza bene.
- No. Dorme male.
- No. Dorme molto male.

74. Come definirebbe l'umore del bambino quando si sveglia il mattino?

- Di ottimo umore
- Di buon umore
- Normale
- Piuttosto di cattivo umore
- Di pessimo umore

75. In una giornata "standard", quanti pisolini presenta generalmente il bambino in orario DIURNO? Indicare il NUMERO complessivo di pisolini che si osservano tra il risveglio del mattino e il momento di coricarsi la sera.

76. Quanto tempo, COMPLESSIVAMENTE, il Suo bambino dorme IN ORARIO DIURNO in una giornata "standard"? Sommare la durata di tutti i pisolini diurni. Si prega di esprimere il tempo totale in MINUTI (es. 20 minuti; 90 minuti; 150 minuti...).

77. Ritiene che il sonno del Suo bambino rappresenti un problema?

- No, non è per niente un problema.
- Sì, ma è un problema di scarsa rilevanza.
- Sì, è un piccolo problema.
- Sì, è un discreto problema.
- Sì, è un problema serio.

78. Quanto si sente sicuro nella gestione del sonno del Suo bambino?

- Molto sicuro
- Piuttosto sicuro
- Normale
- Piuttosto insicuro
- Molto insicuro

Il sonno...in famiglia

79. I genitori o i fratelli del bambino presentano disturbi del sonno?

- Sì
- No
- Non so

80. Qualora avesse risposto "Sì" alla domanda precedente, specifichi il tipo di Disturbo del sonno che intende segnalare. Sono ammesse più risposte.

- Difficoltà ad addormentarsi
- Sindrome delle gambe senza riposo (Restless leg syndrome)
- Risvegli frequenti nel corso della notte
- Incubi o sogni vividi
- Sonnambulismo
- Parlare nel sonno (sonniloquio)
- Digrignare i denti (bruxismo)
- Altro:

81. A che ora si corica abitualmente la sera? Indicare ora (da 00 a 23) e minuti (da 00 a 59).

82. A che ora si sveglia abitualmente il mattino? Indicare ora (da 00 a 23) e minuti (da 00 a 59).

83. Al risveglio del mattino, si sente di solito sufficientemente riposato?

- Sì
- No

Terapie farmacologiche in corso

84. Il bambino assume farmaci come terapia cronica? NON consideri qui eventuali supplementazioni vitaminiche (es. vitamina D, vitamina K).

- Sì
- No

85. Se il bambino assume una terapia farmacologica cronica, questi farmaci sono assunti da più di 10 giorni?

- Sì, il bambino assume farmaci da più di 10 giorni
- Sì, il bambino assume farmaci ma la terapia è in corso da meno di 10 giorni
- Il bambino NON assume alcuna terapia cronica

86. Se il bambino assume una terapia farmacologica cronica, tra i farmaci in terapia è compreso il propranololo?

- Sì
- No

Terapia con propranololo

87. A che ora il Suo bambino assume abitualmente la prima dose quotidiana del farmaco? Indicare ora (da 00 a 23) e minuti (da 00 a 59).

88. A che ora il Suo bambino assume abitualmente la seconda dose quotidiana del farmaco? Indicare ora (da 00 a 23) e minuti (da 00 a 59).

89. A prescindere dalla malattia per cui il bambino assume il farmaco, ritiene che qualcosa si sia modificato dopo l'introduzione della terapia con propranololo? NON consideri qui eventuali cambiamenti relativi alla patologia di base.

- No, non ho rilevato nessun cambiamento.
- Sì, ci sono stati piccoli cambiamenti.
- Sì, ci sono cambiamenti rilevanti.
- Non so

Dopo la terapia... qualcosa è cambiato?

90. Qualora avesse notato cambiamenti con la terapia e indipendentemente da eventuali modifiche della patologia di base, cosa ritiene sia cambiato nel Suo bambino dopo l'introduzione della terapia con propranololo? Sono ammesse più risposte.

- NON si sono manifestati cambiamenti di alcun tipo.
- Si sono verificati episodi di broncospasmo e/o altri disturbi respiratori
- Si sono verificati episodi di debolezza associati a sudorazione abbondante e/o scarsa reattività
- Si sono verificati episodi di diarrea in assenza di vomito e/o febbre
- Si sono verificati cambiamenti del sonno (in orario notturno e/o diurno)
- Si sono verificate Irritabilità e/o agitazione in orario diurno
- Si è verificata sonnolenza eccessiva in orario diurno
- Si è verificato altro tipo di cambiamento

91. Se nella risposta alla domanda precedente ha indicato l'opzione "Altro tipo di cambiamento", specifichi cosa ha rilevato.

92. Qualora avesse rilevato cambiamenti dopo l'introduzione della terapia con propranololo, indichi per favore l'età del bambino in MESI in cui questi cambiamenti si sarebbero manifestati. Se non si sono rilevati cambiamenti, lasci la risposta in bianco.

93. In relazione ad eventuali cambiamenti da Lei segnalati, sono state apportate modifiche alla terapia?

- No, La terapia non è stata modificata perché non ho rilevato alcun disturbo.
- No, la terapia non è stata modificata perché non avevo ritenuto necessario segnalare al Medico i cambiamenti suddetti.
- No, la terapia non è stata modificata nonostante avessi segnalato i cambiamenti suddetti.
- Sì, la terapia è stata interrotta definitivamente.
- Sì, la terapia è stata interrotta transitoriamente e poi ripresa.

Dopo la terapia... Disturbi respiratori

94. A seguito dell'introduzione della terapia con propranololo, ha rilevato cambiamenti per quanto attiene ad eventuali disturbi respiratori? Considerare SOLO sintomi come tosse persistente, broncospasmo, respiro sibilante, ritmo respiratorio accelerato rispetto all'abituale, difficoltà respiratoria, pianto debole, facile affaticamento (ad esempio, con difficoltà ad alimentarsi).

- Non vi sono mai stati disturbi respiratori (né in passato, né dopo terapia). → Passa alla domanda 103.
- Sono comparsi disturbi respiratori che non si erano mai verificati in precedenza.
- Vi erano già disturbi respiratori e questi, dopo la terapia, sono rimasti invariati.
- Vi erano già disturbi respiratori e questi, dopo la terapia, sono peggiorati.
- Vi erano già disturbi respiratori e questi, dopo la terapia, sono migliorati.

95. Se si sono verificati problemi respiratori dopo la terapia con propranololo, il bambino presentava contemporaneamente raffreddore, febbre o altri sintomi "simil-influenzali"?

- Si sono verificati problemi respiratori dopo la terapia, ma non ne ricordo le caratteristiche.
- Alcune volte gli episodi erano associati a febbre o altri sintomi simil-influenzali, altri no.
- Tutti gli episodi erano SENZA febbre o altri sintomi simil-influenzali.
- Tutti gli episodi erano CON contestuale febbre e/o sintomi simil-influenzali.

96. Se si sono verificati problemi respiratori, indichi per favore QUANTI episodi di disturbo respiratorio (es. tosse persistente, broncospasmo, respiro sibilante, ritmo respiratorio accelerato, difficoltà respiratoria, pianto debole, facile affaticamento) si sono verificati. Riportare il numero di episodi e l'intervallo temporale considerato. Esempio: "3 episodi in 3 mesi", "2 episodi in 6 mesi", "1 episodio in 1 anno"...

97. Qualora i disturbi respiratori segnalati fossero comparsi ex novo oppure peggiorati dopo l'introduzione della terapia, dopo quanto tempo dall'introduzione della terapia ritiene siano insorti questi cambiamenti? Indicare l'intervallo temporale in SETTIMANE.

98. Ritiene che il disturbo respiratorio del Suo bambino rappresenti un problema?

- Il mio bambino ha alcuni disturbi respiratori ma non è per niente un problema.

- Il mio bambino ha alcuni disturbi respiratori, ma è un problema di scarsa rilevanza.
- Il mio bambino ha alcuni disturbi respiratori ma è un piccolo problema.
- Il mio bambino ha alcuni disturbi respiratori e questo è un discreto problema.
- Il mio bambino ha alcuni disturbi respiratori e questo è un problema serio.

99. Qualora fossero segnalati disturbi respiratori dopo l'introduzione della terapia, si è reso necessario un utilizzo più frequente di farmaci con aerosol contenente Salbutamolo o Ipratroprio oppure puff RISPETTO AL PERIODO PRE-TERAPIA con propranololo?

- Sì, vi sono disturbi respiratori ma l'utilizzo di questi farmaci è rimasto invariato per frequenza rispetto al passato.
- Sì, vi sono disturbi respiratori e questi farmaci sono stati utilizzati con maggiore frequenza rispetto al passato.
- Sì, vi sono disturbi respiratori e questi farmaci sono stati utilizzati con minore frequenza rispetto al passato.

100. I problemi respiratori che Lei ha segnalato dopo terapia con propranololo hanno richiesto ricovero del bambino in ospedale? Considerare come "ricovero" la necessità di permanere in ospedale per almeno 24 ore.

- Ci sono stati problemi respiratori dopo l'introduzione di terapia, ma NESSUN ricovero per questo motivo.
- Ci sono stati problemi respiratori dopo l'introduzione di terapia, con UN SOLO ricovero per questo motivo.
- Sono stati necessari DUE O PIU' ricoveri a causa dei disturbi respiratori insorti dopo terapia con propranololo.

101. In relazione ai disturbi respiratori eventualmente segnalati, sono state apportate modifiche alla terapia con propranololo?

- Sì, la terapia è stata interrotta definitivamente.
- Sì, la terapia è stata interrotta transitoriamente e poi ripresa.
- No, la terapia non è stata modificata.

102. Qualora la terapia fosse stata modificata a causa di interferenze con eventuali disturbi respiratori del bambino, ritiene che la modifica terapeutica abbia influito in maniera evidente su questi disturbi nel periodo successivo? Qualora non fossero state apportate modifiche alla terapia, lasci la risposta in bianco.

- Il disturbo respiratorio non è cambiato dopo la modifica di terapia.
- Il disturbo respiratorio è migliorato dopo la modifica di terapia.
- Il disturbo respiratorio è peggiorato dopo la modifica di terapia.
- Non so
- Non sono state attuate modifiche di terapia.

Dopo la terapia... Il sonno

103. A seguito dell'introduzione della terapia con propranololo, ha rilevato in particolare cambiamenti per quanto attiene al sonno del Suo bambino?

- Sì

- No
- Non so

104. Qualora avesse osservato cambiamenti nel sonno del Suo bambino a seguito della terapia continuativa con propranololo, indichi per favore quali disturbi ha rilevato. Sono ammesse più risposte.

- Non ho rilevato cambiamenti.
- Si sono verificate irritabilità e/o agitazione in orario diurno
- Si sono verificate irritabilità e/o agitazione in orario notturno
- Si sono verificati episodi di pianto inconsolabile in orario notturno
- Si sono verificati incubi e/o risvegli improvvisi con pianto
- Si sono verificate sonnolenza e/o stanchezza eccessiva in orario diurno
- Si è verificata difficoltà all'addormentamento in orario diurno
- Si è verificata difficoltà all'addormentamento in orario notturno

105. Qualora avesse osservato cambiamenti nel sonno del Suo bambino a seguito della terapia con propranololo, indichi se sono state apportate modifiche alla terapia.

- No, la terapia non è stata modificata perché non ho rilevato cambiamenti.
- No, la terapia non è stata modificata perché non ho ritenuto necessario segnalare questo cambiamento al Medico.
- No, la terapia non è stata modificata nonostante avessi segnalato questo cambiamento al Medico.
- Sì, la terapia è stata interrotta transitoriamente e poi ripresa.
- Sì, la terapia è stata interrotta definitivamente.

106. Qualora la terapia fosse stata modificata a causa di interferenze con il sonno del Suo bambino, ritiene che la modifica terapeutica abbia influito in maniera evidente sul sonno nel periodo successivo?

- Sì. Il sonno è migliorato dopo la modifica di terapia.
- No. Il sonno non è cambiato dopo la modifica di terapia.
- Non so
- La terapia non è stata modificata.
